# Protein Profiling of Preeclampsia Placental Tissues

**DOI:** 10.1371/journal.pone.0112890

**Published:** 2014-11-13

**Authors:** Chang Shu, Zitao Liu, Lifeng Cui, Chengguo Wei, Shuwen Wang, Jian Jenny Tang, Miao Cui, Guodong Lian, Wei Li, Xiufen Liu, Hongmei Xu, Jing Jiang, Peng Lee, David Y. Zhang, Jin He, Fei Ye

**Affiliations:** 1 Department of Obstetrics and Gynecology, The First Hospital of Jilin University, Changchun, Jilin, China; 2 Department of Obstetrics and Gynecology, Tufts Medical Center, Tufts University School of Medicine, Boston, Massachusetts, United States of America; 3 Department of Medicine Bioinformatics Core, Icahn School of Medicine at Mount Sinai, New York, New York, United States of America; 4 Computer Center of Jilin Province, Changchun, Jilin, China; 5 Department of Obstetrics, Gynecology and Reproductive Science, Icahn School of Medicine at Mount Sinai, New York, New York, United States of America; 6 Department of Gastrointestinal Surgery, Shandong Provincial Hospital, Jinan, Shandong, China; 7 Department of Gastrointestinal Surgery, The First Hospital of Jilin University, Changchun, Jilin, China; 8 Department of Ophthalmology, The First Hospital of Jilin University, Changchun, Jilin, China; 9 Division of Clinical Epidemiology, The First Hospital of Jilin University, Chang chun, Jilin, China; 10 Departments of Pathology, Urology and New York University Cancer Institute, New York University, School of Medicine, New York, New York, United States of America; 11 Department of Pathology, Icahn School of Medicine at Mount Sinai, New York, New York, United States of America; International Centre for Genetic Engineering and Biotechnology, Italy

## Abstract

Preeclampsia is a multi-system disorder involved in pregnancy without an effective treatment except delivery. The precise pathogenesis of this complicated disorder is still not completely understood. The objective of this study is to evaluate the alterations of protein expression and phosphorylations that are important in regulating placental cell function in preterm and term preeclampsia. Using the Protein Pathway Array, 38 proteins in placental tissues were found to be differentially expressed between preterm preeclampsia and gestational age matched control, while 25 proteins were found to be expressed differentially between term preeclampsia and matched controls. Among these proteins, 16 proteins and their associated signaling pathways overlapped between preterm and term preeclampsia, suggesting the common pathogenesis of two subsets of disease. On the other hand, many proteins are uniquely altered in either preterm or term preeclampsia and correlated with severity of clinical symptoms and outcomes, therefore, providing molecular basis for these two subsets of preeclampsia. Furthermore, the expression levels of some of these proteins correlated with neonatal small for gestational age (PAI-1 and PAPP-A) and adverse outcomes (Flt-1) in women with preterm preeclampsia. These proteins could potentially be used as candidate biomarkers for predicting outcomes of preeclampsia.

## Introduction

Preeclampsia is characterized by the new onset of hypertension and either proteinuria or end-organ dysfunction after 20 weeks of gestation in previously normotensive women. It occurs in 2–8% of pregnancies, resulting in substantial maternal and neonatal mortality and morbidity worldwide [1]–[3]. Based on the gestational age at the time of clinical recognition of disease, it may be subclassified as preterm preeclampsia and term preeclampsia. Both of them share the common presenting features such as hypertension, proteinuria or end-organ dysfunction, however, they also demonstrate different maternal and fetal outcomes, clinical features, biochemical markers, heritability and the underlying molecular pathogenesis [4], [5]. Preterm preeclampsia occurs before 37 weeks of gestation and commonly represents placental dysfunction, reduction in placental volume, intrauterine growth restriction, abnormal uterine and umbilical artery, low birth weight, perinatal death, and a high incidence of the life-threatening complications to the mother [6], [7]. In contrast, the term preeclampsia, which occurs after 37 weeks of gestation, has mild clinical presentations and more favorable maternal and neonatal outcomes.

The involvement of the abnormal placenta in the pathogenesis of both forms of preeclampsia is unquestionable, but the mechanisms that finally trigger the disease at different gestational age are still not fully elucidated. Although abnormal histopathological findings can be detected in placentas from women with term preeclampsia, pronounced placental morphological abnormalities are mainly found in preterm preeclampsia [8], [9]. It is believed that placental ischemia-reperfusion injury leading to oxidative stress is the trigger for the increased placental release of apoptotic-necrotic trophoblast debris, pro-inflammatory cytokines and anti-angiogenic factors, which cause maternal generalized endothelial cell dysfunction and an exaggerated systemic inflammatory response [2].

The rapid development of gene microarray and next generation sequencing has expedited the understanding of the genetic landscape of preeclampsia [10]–[12]. However, due to the lack of effective tools to investigate a large number of the protein expression, the pattern of protein expression and modification in preeclampsia placenta tissue has not been reported previously. In this study, we investigated the expression of 167 proteins and phosphoproteins in placental tissues from preterm and term preeclampsia as well as gestational age matched controls by means of Protein Pathway Array (PPA) method, a multiplex immunoblot-based assay combined with computational analysis [13]. The Protein Pathway Array is a proteomic method that can characterize hundreds of proteins in tissue samples and identify alterations in protein expression. Our current study aims to reveal the similarities and differences in the placental proteins and signal pathways between preterm and term preeclampsia as well as between preeclampsia and normal pregnancy in order to understand the underlying mechanisms involved in these two forms of preeclampsia. Our goal is to find novel biomarker candidates in the screening of preterm and term preeclampsia or stratify the risks of preterm preeclampsia.

## Materials and Methods

### Patient characteristics

166 pregnant women treated at the Department of Obstetrics, the First Hospital of Jilin University, Changchun, China between July, 2011 and June, 2012 were enrolled in this study and were divided into the following groups based on the gestational age: (1) preterm preeclampsia (<37 weeks), (2) term preeclampsia (≥37 weeks), (3) preterm (<37 weeks) controls, and (4) term (≥37 weeks) controls. In order to avoid the effect of labor on placenta, only the patients delivered by elective cesarean section before the onset of labor were enrolled in this study. Patients complicated with multiple gestations, fetal congenital malformation, fetal chromosomal disorders, or maternal history of cardiovascular, renal, or other hypertension-associated diseases were excluded from the current study. The term and preterm controls are the patients who delivered neonates with birth weight appropriate for gestational age and had no fetal and maternal medical complications or suspected perinatal infections.

The diagnoses of preeclampsia were based on modified American College of Obstetricians and Gynecologists criteria [14]. Preeclampsia in this study was defined as a pregnancy complicated with new onset of hypertension (blood pressure≥140/90 mm Hg on 2 occasions 2 hours to 2 weeks apart after 20 weeks of gestation and proteinuria (urine dipstick with ≥2+ protein at presentation, or 24-hour urine protein ≥300 mg/d).

Adverse maternal outcomes were defined as the presence of new onset of hypertension during the pregnancy plus one of the following: elevated alanine aminotransferase (ALT≥80 U/L), platelet count ≤100×10^9^/L, disseminated intravascular coagulation, abruption, pulmonary edema, cerebral hemorrhage, eclampsia, acute renal failure (creatinine>114.4 µmol/L), or maternal death [15], [16]. The adverse fetal/neonatal outcomes in preeclampsia group included iatrogenic delivery indicated for hypertensive complications of pregnancy as reported by the primary obstetrician, small-for-gestational-age birth weight (≤10th percentile for gestational age), abnormal umbilical artery Doppler findings (absent or reverse flow), fetal death, and neonatal death [15], [16]. The research protocol was approved by the ethics committee of Jilin University's Institution Ethical Review Boards and written informed consent was obtained. The demographics and clinical characteristics of the patients were summarized in **Table 1**.

**Table 1:** Demographic and clinical characteristics of the study groups.

Characteristic	Preterm controls	Preterm preeclampsia	Term Controls	Term preeclampsia
	(n = 36)	(n = 71)	(n = 29)	(n = 30)
**Women**				
Age (yr)	27.0±4.9	27.3±5.43	31.0±5.3	26.6±6.5††
Height (m)	162.7±3.4	161.3±3.3	162.2±5.5	159.4±4.4†
Weight (kg)	72.5±2.7	79.1±4.0***	75.4±2.8	76.3±3.5
Body Mass Index	27.5±1.3	30.4±1.8***	28.7±1.4	30.1±2.0††
Nulliparous - no. (%)	32(88.9)	57(80.3)	21(72.4)	27(90)
Highest SBP at Triage (mmHg)	124.2±13.3	173.0±20.8***	117.4±7.2	162.1±23.0†††
Highest DBP at Triage (mmHg)	80.7±12.8	116.3±15.6***	77.9±7.7	105.8±15.0†††
Gestational age at Triage (wks)	32.9±3.0	33.2±3.0	38.6±1.0	38.5±1.7
Gestational age at delivery (wks)	33.8±2.4	33.7±2.7	39.0±1.0	38.7±1.4
Proteinuria - no. (%)	0	71(100%)***	0	30(100%)†††
ALT at Triage (U/L)	22.4±37.2	45.4±95.0	10.5±5.6	19.5±21.7†
Creatinine at Triage (umol/L)	40.1±7.0	74.6±30.2***	45.2±7.2	61.6±15.6†††
Uric Acid at Triage (umol/L)	164.6±15.0	373.6±87.1***	180.4±40.0	298.6±70.1†††
Platelet Count at Triage (×10^9^/L)	226.7±54.4	161.0±74.3***	203.9±56.3	197.3±69.0
**Infants**				
Birth weight (g)	2157.3±529.5	1893.7±638.0 *	3471.4±461.5	2940.7±828.2†††
Delivery<37wk - no. (%)	36(100%)	71(100%)	0	0
**Placenta**				
Placenta weight (g)	503.6±120.6	453.4±99.5*	599.7±65.1	531.7±71.3†††
**Any Adverse Outcome - no. (%)**	0	48 (67.6)	0	6 (20)
**Maternal Adverse Outcome**				
HTN+Abnormal LFTs/Platelets - no. (%)	0	13 (18.3)	0	1 (3.3)
HTN+DIC - no. (%)	0	1 (1.4)	0	1 (3.3)
HTN+Abruption - no. (%)	0	11 (15.5)	0	2 (6.7)
HTN+Pulmonaryedema - no. (%)	0	4 (5.6)	0	2 (6.7)
HTN+Eclampsia - no. (%)	0	10 (14.1)	0	1 (3.3)
**Fetal and Neonatal Adverse Outcome**				
Fetal Death - no. (%)	0	4 (5.6)	0	2 (6.7)
Neonatal Death - no. (%)	0	7 (9.9)	0	0
Small for gestational age (<10thpercentil) - no. (%)	0	34 (47.9)	0	5 (16.7)

SBP = systolic blood pressure, DBP = diastolic blood pressure, ALT = alanine transaminase, DIC = Disseminated Intravascular Coagulation

Plus–minus values are means±SD.

*P<0.05 compared with gestational age matched, preterm controls;

**P<0.01 compared with gestational age matched, preterm controls;

***P<0.001 compared with gestational age matched, preterm controls.

† P<0.05 compared with gestational age matched, term controls;

†† P<0.01 compared with gestational age matched, term controls;

††† P<0.001 compared with gestational age matched, term controls.

### Placental tissue sampling

All of the placental tissues were obtained within 10 min after Caesarean sections and collected from macroscopically normal areas excluding sites of infarction, hemorrhage and calcification. A specimen of placental tissue (1 cm × 1 cm × 1 cm) was collected near the maternal side of the placenta around the umbilical cord in a sterile condition. Then the specimen was cleansed with sterile normal saline to remove the contaminated blood and amniotic fluid, the tissues were immediately frozen in liquid nitrogen and stored until use.

### Protein Pathway Array Analysis

Total protein was extracted from the 166 fresh frozen placental samples as previously described [13].Briefly, one mL of 1× lysis buffer (Cell Signaling Technology, Danvers, MA) with 1× protease inhibitor cocktail (Roche Applied Science, Indianapolis, IN) and 1× phosphatase inhibitor cocktail (Roche Applied Science, Indianapolis, IN) were added to each tissue sample and the lysate was sonicated 3 times for 15 seconds each time on ice water, then centrifuged at 14,000 rpm for 30 min at 4°C to remove incompletely lysed debris. The protein concentration was determined using BCA Protein Assay kit (PIERCE, Rockford, IL). Three hundred µg of lysated protein was loaded in one well across the entire width of 10% SDS polyacrylamide and separated by electrophoresis [17]. After electrophoresis, the protein was transferred electrophoretically to a nitrocellulose membrane (Bio-Rad, Hercules, CA). Three nitrocellulose membranes were made from each sample and each membrane was blocked for 1 hour with blocking buffer including 3% BSA in 1× TBST containing 20 mMTris-HCl (pH 7.5), 100 mMNaCl, and 0.1% Tween-20. The membrane was then clamped on a Western blotting manifold (Mini-PROTEAN II Multiscreen apparatus, Bio-Rad, Hercules, CA) which isolates 20 channels across the membrane. The multiplex immunoblot was performed using a total of 167 protein-specific or phosphorylation site-specific antibodies (**Table S1**: **List of antibodies included in the Protein Pathway Array**). The antibody library was built basing on our previous research and literature review. Six sets of antibodies (a total of 30–36 antibodies in the first four sets and 18 protein-specific antibodies in the last two sets) were individually applied for each membrane. After overnight incubation at 4°C with primary antibodies, the membrane was washed with 1× TBS and 1× TBST and was then incubated with secondary anti-rabbit (Bio-Rad, Hercules, CA) or anti-mouse (Bio-Rad, Hercules, CA) or anti-goat (Santa Cruz Biotechnology, Santa Cruz, CA) antibody conjugated with horseradish peroxidase for 1 hour at room temperature. The membrane was developed with chemiluminescence substrate (Immun-Star HRP Peroxide Buffer/Immun-Star HRP Luminol Enhancer) (Bio-Rad,Hercules, CA), and chemiluminescent signals were captured using the ChemiDoc XRS System (Bio-Rad, Hercules, CA). The same membrane was then stripped off using stripping buffer (Restore Western blot stripping buffer, Thermo Scientific, Rockford, 1L) and then used for blotting with another set of primary antibodies as described above. Each membrane was blotted for three times.

For Protein Pathway Array data analysis, the signal of each protein were determined by densitometric scanning (Quantity One software package, Bio-Rad) and the background was locally subtracted from raw protein signal. The background-subtracted intensity was normalized by “global median subtraction” method to reduce variation among different experiments, that means, the intensity of each protein from each sample divided by total intensities of all proteins from the same sample and then multiplied by average intensities of all proteins in all samples [18].

### Statistical Analysis

Class comparison analysis was performed using unpaired Student's *t* test to identify proteins differentially expressed between groups. Bivariate Spearman correlation test was used to analyze the correlation between differentially expressed proteins and preterm or term preeclampsia severity. If the R value of placenta proteins correlated with preeclampsia was>0.4, these proteins were further analyzed using One-way analyses of variance (ANOVA) with Post Hoc Multiple Comparisons between subgroups. In order to calculate the association of preterm preeclampsia with adverse outcome and neonatal small-for-gestational age, univariate and multivariate logistic regression analyses were performed. The analyses generated a set of independent factors with corresponding regression coefficient, *P* value, odds ratio (OR) and 95% confidence interval (IC) of the OR. The calibration of the model that describes association between placental specific protein and neonatal small-for-gestational age or adverse outcomes in preeclampsia was performed using the Hosmer-Lemeshow goodness-of-fit test. The logistic regression was performed using SPSS 17.0 software package (SPSS, Chicago, IL). A *P* value <0.05 was considered to be statistically significant.

### Signaling network analysis

The discriminating genes of the corresponding proteins identified by PPA were imported into IPA (Version 9.0, www.ingenuity.com) for network analysis. IPA uses the Ingenuity Knowledge Base as a reference set and identifies local networks that are particularly enriched for the input genes by computational algorithms. Furthermore, IPA uses a Fisher's exact test to determine which pathways (i.e. canonical pathways and biological functions) are significantly linked to the input gene set compared with the whole Ingenuity knowledge base. This analysis was to attempt to explore the different signaling pathways in placenta tissues between preterm or term preeclampsia and gestational age matched control patients.

## Results

### Clinical characteristics

Clinical characteristics of the 166 subjects are summarized in **Table 1**. As expected, women with preterm preeclampsia and term preeclampsia collectively presented proteinuria, significantly elevated uric acid, Body Mass Index (BMI), creatinine and systolic and diastolic blood pressures, decreased platelet count in triage, comparing to the gestational age matched control group (*P*<0.01). Neonatal birth weight and placental weight was significantly higher in control group of women with uncomplicated pregnancies compared to preeclampsia groups (*P*<0.05). 34 out of 71 (47.9%) infants from preterm preeclampsia patients were small for gestational age. In contrast, 5 out of 30 (16.7%) infants from term preeclampsia patients were small for gestational age. There was no difference in gestational age in triage, gestational age at delivery and parity between preterm preeclampsia or term preeclampsia and gestational age matched control groups. Overall, the gestational age at delivery for preeclampsia women with associated small for gestational age was slightly lower than the other groups. Adverse outcomes occurred in 20% of term preeclampsia patients (n = 30) but 67.6% of preterm preeclampsia patients (n = 71).

### Differentially expressed proteins in preterm or term preeclampsia

In order to identify the differentially expressed proteins and phosphoproteins in placenta, the expression levels of each protein between 71 preterm preeclampsia and 36 preterm control placentas, or between 30 term preeclampsia and 29 term controls were compared. Among the 167 proteins and phosphoproteins tested, 64 proteins were detected in either control or preeclampsia placentas **(Table S1)**. Of these proteins, 38 showed significant differences between preterm preeclampsia and preterm controls (*P*<0.05), including 20 up-regulated proteins and 18 down-regulated proteins in preterm preeclampsia. On the other hand, 25 proteins and phosphoproteins showed significant differences between term preeclampsia and term controls (*P*<0.05), including 16 up-regulated proteins and 9 down-regulated proteins in preterm preeclampsia (**Table 2** **)**.

**Table 2:** Differentially expressed proteins between preterm preeclampsia or term preeclampsia and gestational age matched controls.

Preterm PE vs Preterm Con			Term PE vs Term Con		
Up-regulation (n = 20)	Fold Change	P value	Up-regulation (n = 16)	Fold Change	P value
**Calretinin**	**2.6**	**1.6E-13**	**Calretinin**	**2**	**8.9E-07**
**HtrA**	**4.6**	**8.1E-11**	**HtrA**	**3.7**	**1.0E-02**
**Nox4**	**1.6**	**5.0E-08**	**Nox4**	**1.5**	**7.9E-04**
**Flt-1**	**5.8**	**3.0E-08**	**Flt-1**	**2.5**	**2.7E-03**
**HSP90**	**2.3**	**3.1E-07**	**HSP90**	**1.9**	**7.9E-03**
**Ptx3**	**2.2**	**2.0E-05**	**Ptx3**	**1.8**	**1.5E-02**
**Endoglin**	**1.3**	**6.7E-04**	**Endoglin**	**1.3**	**5.9E-03**
**TFPI2**	**1.4**	**3.4E-03**	**TFPI2**	**1.6**	**1.5E-02**
**Ebi3**	**1.8**	**9.1E-03**	**Ebi3**	**3.1**	**7.3E-03**
**PSM**	**1.3**	**8.2E-03**	**PSM**	**1.6**	**6.1E-04**
**PBEF**	**1.6**	**3.5E-02**	**PBEF**	**1.8**	**2.2E-02**
PAI1	3	8.8E-09	p-PKCδ	1.8	3.0E-04
Factor XIII B	1.7	3.1E-05	PAF	1.4	4.3E-03
EGFR	1.3	5.0E-05	AKT	1.3	2.8E-02
CD55	2.2	6.2E-05	PIGS	1.7	2.8E-02
ALP	2.4	8.6E-04	p-PKC α/βII	1.8	3.5E-02
Vimentin	1.6	1.6E-04			
p22-phox	1.3	4.0E-03			
Stat1	1.6	4.7E-03			
WT1	1.4	8.1E-03			
**Down-regulation (n = 18)**	**Fold Change**	**P value**	**Down-regulation (n = 9)**	**Fold Change**	**P value**
**Annexin XI**	**−2.3**	**1.2E-09**	**Annexin XI**	**−1.9**	**6.0E-05**
**PIGF**	**−1.9**	**3.4E-07**	**PIGF**	**−1.9**	**4.0E-06**
**p-MAPK(Erk1/2)**	**−2.4**	**1.9E-05**	**p-MAPK(Erk1/2)**	**−1.7**	**4.6E-02**
**Bcl-6**	**−2**	**1.5E-04**	**Bcl-6**	**−1.6**	**6.2E-03**
**Glypican3**	**−1.5**	**3.0E-03**	**Glypican3**	**−1.3**	**4.6E-02**
PDEF	−1.8	4.8E-13	TFIIH p89	−1.3	1.2E-03
CREB	−1.7	5.2E-06	Bcl-2	−1.6	1.5E-02
NFkB p65	−1.7	2.5E-06	p-Stat3	−1.5	2.5E-02
ICAM-1	−1.2	3.2E-04	nrf2	−1.3	4.5E-02
cdc25B	−1.2	3.7E-04			
14-3-3β	−1.5	4.9E-04			
PCNA	−1.7	7.7E-04			
Bcl-xL	−1.2	5.9E-04			
Notch4	−1.5	2.4E-03			
IGFBP3	−1.3	7.5E-03			
PAPP-A	−1.3	1.5E-02			
MetRS	−1.6	2.3E-02			
p-PKC α/βII	−1.5	3.2E-02			
**PIGF**	**−1.9**	**3.4E-07**	**PIGF**	**−1.9**	**4.0E-06**
**p-MAPK(Erk1/2)**	**−2.4**	**1.9E-05**	**p-MAPK(Erk1/2)**	**−1.7**	**4.6E-02**
**Bcl-6**	**−2**	**1.5E-04**	**Bcl-6**	**−1.6**	**6.2E-03**
**Glypican3**	**−1.5**	**3.0E-03**	**Glypican3**	**−1.3**	**4.6E-02**
PDEF	−1.8	4.8E-13	TFIIH p89	−1.3	1.2E-03
CREB	−1.7	5.2E-06	Bcl-2	−1.6	1.5E-02
NFkB p65	−1.7	2.5E-06	p-Stat3	−1.5	2.5E-02
ICAM-1	−1.2	3.2E-04	nrf2	−1.3	4.5E-02
cdc25B	−1.2	3.7E-04			
14-3-3β	−1.5	4.9E-04			
PCNA	−1.7	7.7E-04			
Bcl-xL	−1.2	5.9E-04			
Notch4	−1.5	2.4E-03			
IGFBP3	−1.3	7.5E-03			
PAPP-A	−1.3	1.5E-02			
MetRS	−1.6	2.3E-02			
p-PKC α/βII	−1.5	3.2E-02			

Note: The proteins in bold are the shared ones in both preterm preeclampsia and term preeclampsia groups.

**Figure 1:**
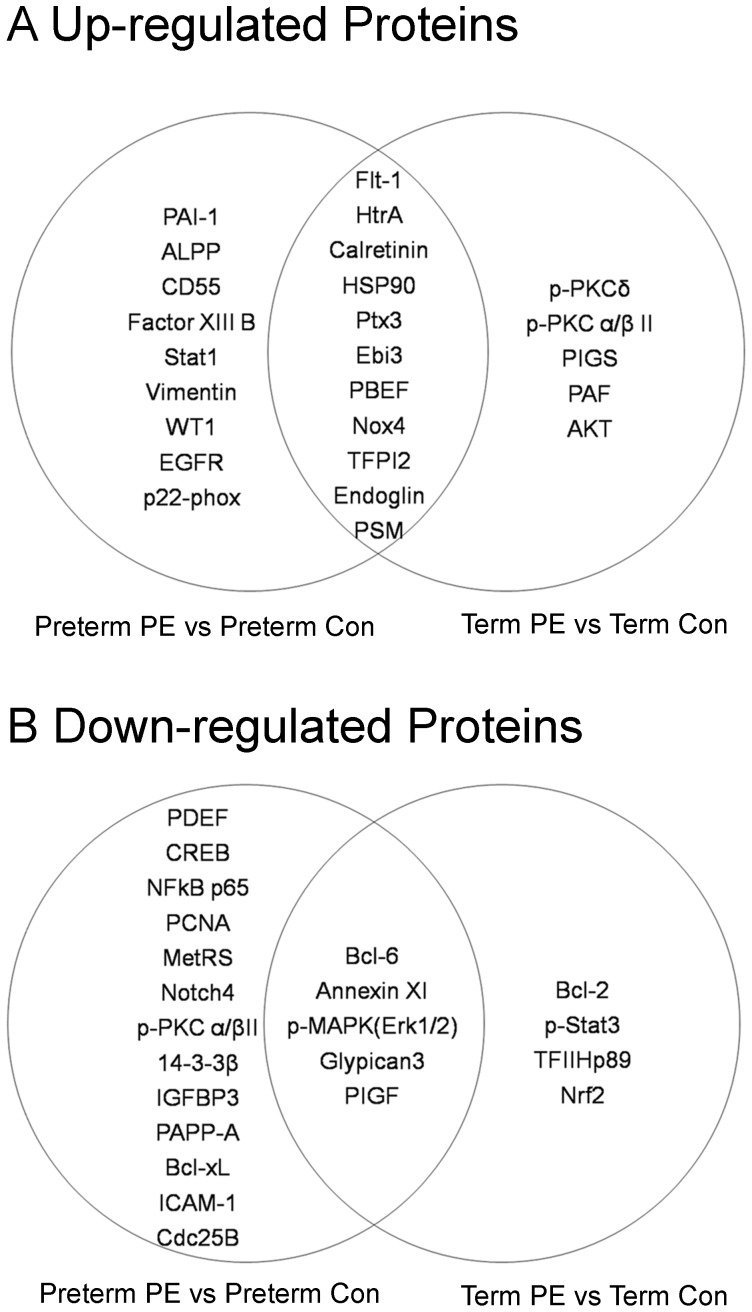
Differentially expressed proteins in placental tissues of preeclampsia. **A.** Comparison of up-regulated proteins in preterm preeclampsia vs. preterm controls **(left circle)** and term preeclampsia vs. term controls **(right circle)**. Overlap between the two circles represent up-regulated proteins in preterm and term preeclampsia. **B**. Comparison of down-regulated proteins in preterm preeclampsia vs. preterm controls **(left circle)** and term preeclampsia vs. term controls **(right circle)**. Overlap between the two circles represent down-regulated proteins in preterm and term preeclampsia.

Of the up-regulated proteins, eleven proteins, including Calretinin, HtrA, Nox4, Flt1, HSP90, Ptx3, Endoglin, TFPI2, Ebi3, PSM, and PDEF were both up-regulated in preterm and term preeclampsia group, indicating their common roles in these two forms of preeclampsia **(** **Figure 1A** **, overlap between two circles)**.PAI-1, Factor XIII B, EGFR, CD55, ALPP, Vimentin, p22-phox, Stat1, and WT1 were differentially expressed in preterm preeclampsia **(** **Figure 1A** **, left circle)**, but not in term preeclampsia. In contrast, 5 proteins were only differentially expressed in term preeclampsia group **(** **Figure 1A** **, right circle)**.

Of the down-regulated proteins, five proteins, including AnnexinXI, PlGF, Bcl-6, p-MAPK(Erk1/2) and Glypican3 were down-regulated in both preterm and term preeclampsia, indicating their common roles in these two forms of preeclampsia **(** **Figure 1B** **, overlap between two circles)**.PDEF, CREB, NF-kB p65, ICAM-1, Cdc25B, 14-3-3β, PCNA, Bcl-xL, Notch4, IGFBP3, PAPP-A, Met-RS, p-PKCα/βII were differentially expressed in preterm preeclampsia **(** **Figure 1B** **, left circle)**, but not in term preeclampsia. In contrast, 4 proteins were only differentially expressed in term preeclampsia group **(** **Figure 1B** **, right circle)**.

### Signaling pathways involved in preterm preeclampsia and term preeclampsia

**Figure 2:**
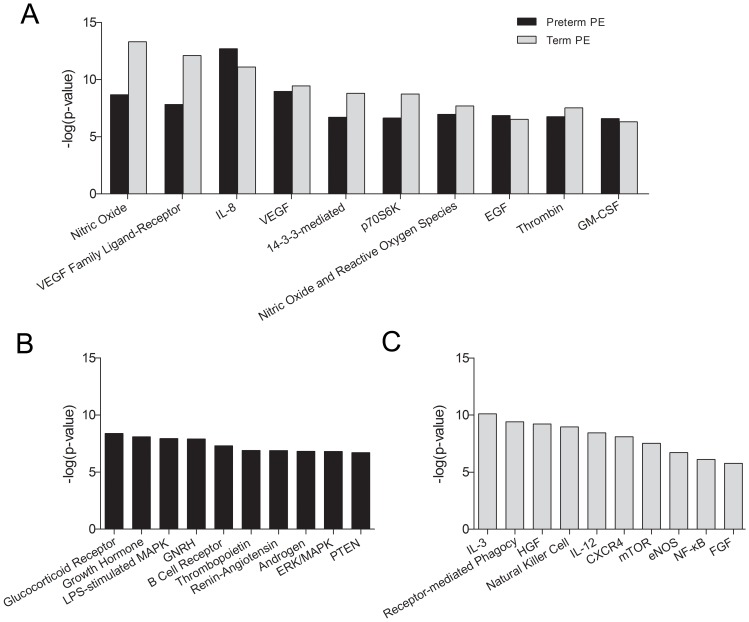
The similarity and difference in signaling pathways altered in term and pre-term preeclampsia. **A**. Common top 10 pathways involved in both preterm and term preeclampsia. **B**. Top 10 pathways only involved in preterm preeclampsia. **C**. Top 10 pathways only involved in term preeclampsia. The length of the bars (-log p-value) indicated the significance of the signaling pathways to the differently expressed proteins related.

To determine signaling pathways affected in preeclampsia placenta tissues, the differentially expressed proteins between preterm or term preeclampsia and gestational age matched controls were input into Ingenuity Pathways Analysis (IPA, www.ingenuity.com). The top 10 canonical signaling pathways shared in both preterm preeclampsia and term preeclampsia were shown in **Figure 2A**, suggesting the common roles of these pathways in preeclampsia. The top 10 canonical signaling pathways only dysregulated in preterm preeclampsia or term preeclampsia were shown in **Figure 2B** and **Figure 2C**, respectively, suggesting the particular canonical signaling pathways involved in either preterm or term preeclampsia.

### Proteins associated with severity of preeclampsia

**Figure 3:**
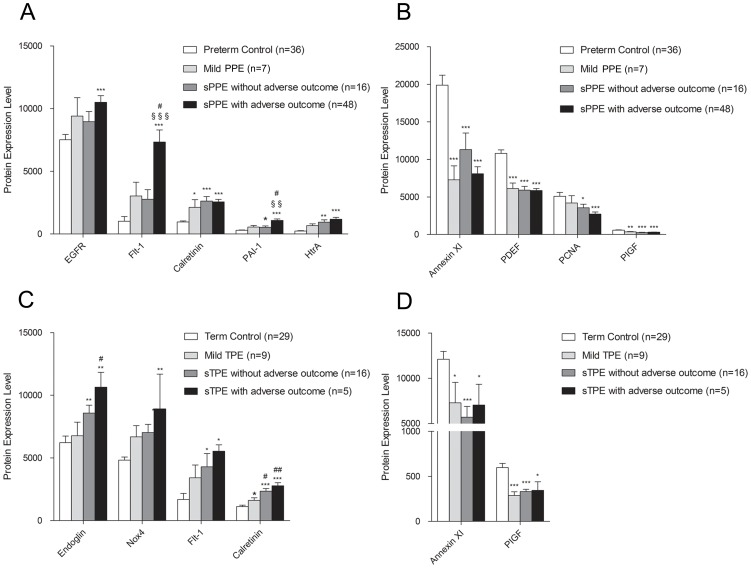
Placental proteins associated with severity of clinical presentations in preeclampsia. The protein expression levels were correlated among 4 groups of pregnant women, either term or preterm delivery, including mild preeclampsia, severe preeclampsia without adverse outcome, severe preeclampsia with adverse outcome as well as gestational age matched controls. **A**. Five proteins positively correlated with severity of preterm preeclampsia. **B**. Four proteins inversely correlated with severity of preterm preeclampsia. **C**. Four proteins positively correlated with severity of term preeclampsia. **D**. Two proteins inversely correlated with severity of term preeclampsia. *P<0.05 compared to controls, **P<0.01 compared to controls, ***P<0.001 compared to controls, #P<0.05 compared to mild preterm or term preeclampsia, ##P<0.01 compared to mild preterm or term preeclampsia, §§ P<0.01 compared to severe preterm or term preeclampsia without adverse outcome. §§§P<0.001 compared to severe preterm or term preeclampsia without adverse outcome. PPE: preterm preeclampsia. sPPE: severe preterm preeclampsia. TPE: term preeclampsia. sTPE: severe term preeclampsia.

In order to determine the relationship between the protein expression and the severity of preeclampsia symptoms, the placenta tissues were separated into four subgroups, including controls, mild preeclampsia, severe preeclampsia without adverse outcomes, and severe preeclampsia with adverse outcomes **(** **Figure 3** **)**.

The bivariate spearman correlation test was used to identify differentially expressed proteins among these subgroups. Among 167 proteins tested, the expression of 5 proteins (EGFR, Flt-1, Calretinin, PAI-1 and HtrA) were positively correlated **(** **Figure 3A** **)** and four proteins (AnnexinXI, PDEF, PCNA and PlGF) were inversely correlated with the severity of preterm preeclampsia (*P*<0.05, R value≥0.4) **(** **Figure 3B** **)**. On the other hand, four proteins (Endoglin, Nox4, Flt-1 and Calretinin) were positively correlated **(** **Figure 3C** **)** and two proteins (AnnexinXI and PlGF) were inversely correlated with the severity of term preeclampsia (*P*<0.05, R value≥0.4) **(** **Figure 3D** **)**. Furthermore, the expression level of Flt-1(*P*<0.001) and PAI-1(*P*<0.01) were significant different in severe preterm preeclampsia women with or without adverse outcome. On the other hand, the expression level of Calretinin was significant different (*P*<0.01) in severe term preeclampsia women with adverse outcome and mild term preeclampsia.

### Proteins associated with small for gestational age in preterm preeclampsia

To examine the relationship between the placental protein expression and the infant weight for gestational age in preterm preeclampsia women, the protein expression levels between small for gestational age group (n = 34) and appreciate for gestational age group (n = 37) were compared using the unpaired student's t-test and five proteins were found to be differentially expressed between two groups. Among these 5 proteins, PAI-1, Flt-1 and Ebi3 were up-regulated, while PSM and 14-3-3β were down-regulated in small for gestational age group. However, a stepwise multivariate logistic regression analysis showed that only PAI-1 (OR 4.065, IC95% 1.872–8.828) and PAPP-A (OR 0.398, IC95% 0.186–0.850) were independent factors associated with small for gestational age in women with preterm preeclampsia **(** **Table 3** **)**.

**Table 3:** Independent protein factors associated with neonatal small for gestational age in preterm preeclampsia.

				95% CI	
Variables	B	*P* value	OR	Lower	Upper
PAI1	1.403	.000	4.065	1.872	8.828
PAPP-A	−0.922	0.017	0.398	0.186	0.850

**Abbreviation: B-regression coefficients; OR-odds ratio; CI-confidence interval.**

### Proteins and clinical factors associated with adverse outcomes in preterm preeclampsia

To identify the placental protein associated with adverse outcomes in patients with preterm preeclampsia, we examined 38 differentially expressed proteins between preterm preeclampsia and preterm controls using univariate logistic regression analysis. The results showed that Flt-1, PAI-1, PBEF and PSM were strongly associated with adverse outcomes in patients with preterm preeclampsia.

Additional logistical regression analysis were performed to determine the clinical factors associated with adverse outcome in women with preeclampsia, which include age, body mass index, nulliparity, highest systolic blood pressure in triage, highest diastolic blood pressure in triage, gestational age in triage, gestational age at delivery, proteinuria, ALT in triage, creatinine in triage, uric acid in triage, platelet count in triage, placenta weight and birth weight. The results showed that highest systolic and diastolic blood pressure in triage, gestational age in triage, gestational age at delivery, proteinuria, ALT in triage, creatinine in triage, uric acid in triage, platelet count in triage, placenta weight and birth weight were associated with the adverse outcome in women with preeclampsia.

Therefore, highest systolic and diastolic blood pressure, gestational age, gestational age at delivery, proteinuria, ALT, creatinine, uric acid, platelet count, placenta weight, birth weight, Flt-1, PAI-1, PBEF and PSM were included in the subsequent stepwise multivariate logistic regression analysis. The results showed that only birth weight, creatinine level in triage and Flt-1 remained as independent risk factors associated with adverse outcome in women with preterm preeclampsia (*P* value  =  0.727 by Hosmer-Lemeshow goodness-of- fit test) **(** **Table 4** **)**.

**Table 4:** Independent factors associated with adverse outcomes in preterm preeclampsia.

				95% CI	
Variables	B	*P* value	OR	Lower	Upper
Birth weight - g	−.002	0.010	0.998	0.997	1.000
Creatinine in Triage- umol/L	.064	0.016	1.066	1.012	1.123
Flt-1	0.454	0.039	1.574	1.024	2.419

**Abbreviation: B-regression coefficients; OR-odds ratio; CI-confidence interval.**

## Discussion

A growing body of evidence suggests that the preterm and term preeclampsia have two distinct pathophysiological processes with distinct maternal and fetal prognosis [19].Furthermore, the different placenta change may be involved in the different pathogenesis of preterm preeclampsia and term preeclampsia. Differences between early and late preeclampsia have been identified in placental gene expression signatures [20]. But less work has been done at the protein level compare preterm preeclampsia and term preeclampsia. Since the maternal circulation protein factors derived from placenta contribute to the main clinical presentations and prognosis in preeclampsia and serve as the biomarkers for diagnosis and monitor the severity of disease as well therapeutic targets, the analysis of placental proteins in preterm and term preeclampsia would be of particular interest.

In this study, we confirmed several placenta proteins known to be changed in preeclampsia. In addition, we also identified several placenta protein expression alterations associated with preeclampsia that have not been previously reported. We utilized Protein Pathway Array to evaluate the expression of 167 proteins in placental tissues of preterm or term preeclampsia and gestational age matched controls. Sixteen proteins were found to be shared by preterm and term preeclampsia. We confirmed several placenta proteins, such as Flt-1 and Endoglin, known to be changed in preeclampsia. In addition, we also identified several placenta proteins whose expression alterations associated with preeclampsia that have not been previously reported. They are involved in multiple signal pathways or functions. These 16 proteins are involved in antiangiogenic factors (sFlt-1, PlGF and sEng), coagulation(TFPI2) [21], apoptosis (Bcl-6), cell differentiation and growth (Glypican3), inflammatory immune response (Ptx3, Ebi3 and PBEF), cell signal [p-MAPK(Erk1/2) and HSP90], protective reactive oxygen species (Nox4), degradation of extracellular matrix (HtrA), Calcium binding protein (Calretinin), Metalloenzyme (PSM), as well as endocytosis and exocytosis cell adhesion (AnnexinXI). These 16 common proteins may be involved in the common pathways leading to the clinical presentations of preterm or term preeclampsia such as hypertension and protein urine. For instance, increased production of antiangiogenic factors (sFlt-1) disturbs the balance of between proangiogenic (VEGF and PlGF) and antiangiogenic factors (sFlt-1) results in the systemic endothelial dysfunction characteristic of preeclampsia [2].

A total of 22 proteins were found to be uniquely dysregulated in preterm preeclampsia compared with preterm control. These differentially expressed proteins involved coagulation (PAI-1, Factor XIII B), apoptosis (Bcl-xL, IGFBP3), DNA repair (PCNA), transcription (CREB, NF-kB p65, PDEF and WT1), cell cycle (Cdc25B), cell adhesion (ICAM-1), cell proliferation (EGFR, PAPP-A), cell differentiation (Notch4), immune response (CD55), cell signal (14-3-3β, Stat1 and p-PKC α/βII), tRNA syntheses (MetRS), ALPP, cytoskeleton (Vimentin) and O_2_ sensors (p22-phox) [22]. Since these 22 dysregulated proteins are particular to the preterm preeclampsia they may contribute to the characters of preterm preeclampsia such as placental dysfunction, reduction in placental volume, intrauterine growth restriction, perinatal death, maternal multi-organ dysfunction, and maternal and neonatal adverse outcomes. Nine proteins were found to be uniquely dysregulated in term preeclampsia as compared with term control. These differentially expressed proteins include anti-apoptosis (Bcl-2), DNA repair (TFIIHp89), transcription (p-Stat3), antioxidant response (Nrf2), cell survival (AKT), cell signal (p-PKC α/βII and p-PKCα), platelet-activating factor (PAF), biosynthesis of the glycosylphosphatidylinositol (GPI)-anchor (PIG-S), suggesting their particular roles in term preeclampsia.

The placenta is the central to the pathogenesis of preeclampsia. It is currently believed that abnormal placentation occurs early in pregnancy and that this leads to placental ischemia (Stage I) in preeclampsia. The ischemic placenta is thought to secrete soluble factors during the third trimester that in turn induce systemic endothelial dysfunction and the maternal syndrome of preeclampsia (Stage II) [19]. The precise trigger of placental ischemia (Stage I) remains to be elucidated. However, several pathways involving deranged vascular, immune and inflammatory response are related with Stage II responses [23]. All of the clinical features of preeclampsia can be explained as clinical responses to generalized endothelial dysfunction [24], [25].

Based on the pathway analysis, our data show that many different pathways are involved in the development and/or presentations of preeclampsia **(** **Figure 2** **)**. Several pathways were shared by preterm and term preeclampsia, suggesting common pathophysiological mechanisms **(** **Figure 2A** **)**. Among them, two VEGF related pathways [26] (VEGF family ligand-receptor interactions, VEGF signaling) and two nitric oxide related pathways [23](nitric oxide signaling, production of nitric oxide and reactive oxygen species) are well-known to cause systemic endothelial dysfunction in preeclampsia. Thrombin signaling enhances sFlt-1 expression in trophoblast [27] and may also function through VEGF pathway in preeclampsia [28]. The involvement of IL-8 signaling and 14-3-3-mediated signaling support the hypothesis that preeclampsia is associated with increased inflammatory response [29], [30]. GM-CSF signaling may be related to immunological abnormalities contributing to the etiology of preeclampsia [31]. The effect of EGF signaling in preeclampsia is exemplified by HB-EGF (heparin binding EGF like growth factor) [32]. The role of p70S6K signaling in preeclampsia has not been reported before. Taking together, it seems that systemic endothelial dysfunction, augmented by excessive inflammatory response, is the major mechanism in the pathogenesis of preeclampsia before delivery.

Since preterm preeclampsia demonstrates protein profile different from term preeclampsia in our study **(** **Figure 1** **)**, we explore the top ten signal pathways specific for preterm preeclampsia **(** **Figure 2B** **)**. MAPK and/or Erk/MAPK signaling is a common pathway downstream to many preeclampsia associated markers or molecules such as stress sensor Gadd45α [33] and heat shock protein 27 (Hsp27) [34], oxidative stress [35] and HB-EGF in invasion and anti-apoptosis upon hypoxia [36], sFlt-1 [37] and angiotensin [38] in maintaining endothelial function, and angiogenic regulator CCN3 [39]. Steroid hormones are involved in the pathogenesis of preterm preeclampsia. Elevated cortisol level in preeclampsia placenta, associated with prematurity and low birth weight [40], suppresses trophoblast proliferation via glucocorticoid receptor [41]. In addition, testosterone and its receptor, androgen receptor, are expressed at increased level in preeclampsia [42]. Increased level of placental growth hormone in serum and amniotic fluid at mid-trimester is an index of intrauterine growth retardation related to preeclampsia [43], and preeclampsia in the third trimester is associated with higher median concentrations of placental growth hormone in both the maternal and fetal circulation compared to normal pregnancy [44]. Renin-angiotensin signaling was found predominantly in preterm preeclampsia in our study, while the previous reports show this deranging systemic endothelial function occurs in both preterm and term preeclampsia [45]. This may be related to our patient population. PTEN may induce sEng release from endothelial cell in maintaining endothelial function [46]. The role of GnRH signal, B cell receptor signal, and thrombopoietin signal in preeclampsia is still unknown. It seems that steroid hormone such as glucocorticoid receptor and androgen signal besides deregulated endothelial function has more remarkable effect in preterm preeclampsia. MAPK signal pathway is one of the common pathways in regulating the pathogenesis of preterm preeclampsia. In future, we will further explore into the mechanism of incomplete spiral artery remodeling and the placental protein factor shedding into the maternal blood based on these top 10 signaling pathways.

In term preeclampsia, IL-3 signaling and natural killer cell signaling which are related with placenta immune response, HGF signaling which is associated with trophoblast invasion [47], FGF signaling which is associated with angiogenesis, and mTOR signaling which regulates the invasive differentiation of human trophoblasts may be involved in the first stage of term preeclampsia. On the other hand, NF-kB signaling, IL-12 signaling, Fcγ receptor-mediated phagocytosis and CXCR4 signaling which contribute to inflammation and eNOS signaling which is associated with oxidative stress may be involved in the second stage of term preeclampsia.

It is worthy to note that more proteins in placenta were dysregulated in preterm preeclampsia than in term preeclampsia, consistent with the more pronounced morphological abnormalities found in early preeclampsia [8], [9]. Furthermore, some of the proteins are strongly associated with the severity of clinical presentation of preeclampsia **(** **Figure 3** **)**. For example, Flt-1, HtrA, Calretinin, PAI-1 and EGFR were positively and PDEF, Annexin XI, PLGF and PCNA were inversely correlated with the severity of preterm preeclampsia. On the other hand, Calretinin, HtrA, Flt-1, Nox4 and Endoglin were positively and Annexin XI and PLGF were inversely correlated with the severity of term preeclampsia. Véronique et al demonstrated that Flt-1 over-expression in the placenta strongly correlates with severity of hypertensive disease [48]. It has been shown that sFlt-1 debris from preeclampsia placenta bind to vascular endothelial growth factors and placental growth factor and deprived the essential survival factors, causing the clinical feature of preeclampsia [2]. Shivalingappa reported the placenta-derived soluble TGF-β co-receptor, Endoglin, which is elevated in the sera of preeclamptic individuals, correlated with disease severity as it can induce vascular permeability and hypertension in vivo [49].

In this study, we showed that PAI-1 and PAPP-A were independent risk factors associated with small for gestational age in women with preterm preeclampsia. Several lines of evidence suggest that dysfunction of the PAI-1 system contributes to the development of pre-eclampsia and/or fetal injury. Plasma PAI-1 level was shown to be increased in patients with overt pre-eclampsia and HELLP syndrome consistent with its anti-invasive and procoagulation role (i.e. increased fibrin deposition in intervillous spaces) [50], [51]. Indeed, in situ studies of pre-eclamptic placenta have also revealed high level of PAI-1 mRNA and protein in placenta [52], [53]. These observations indicate that change in the serum levels of PAI-1 is resulted from its levels of in situ production in preeclampsia placenta. Furthermore, the poor extravillous trophoblast invasion and poor thrombolysis may be apt to form microthrombus in placenta lead to placental dysfunction, hypoxia and nutrition supply defect. The placental bed of patients with preeclampsia with IUGR described by Ivo Bronsens further confirmed our hypothesis. It is characterized by a large number of non-transformed myometrial spiral arteries and such arteries show frequently obstructive lesions such as acute atherosis and thrombosis [54]. The association of PAPP-A with small for gestational age of neonates have been previously reported. Dukhovny et al demonstrated that low PAPP-A was a significant predictor of small for gestational age (OR 3.3, 95% CI 1.5–7.4) [55]. Therefore, serum levels of PAI-1 and PAPP-A could be useful biomarkers to predict fetal development.

In conclusion, our study using innovative proteomic method demonstrated significant alterations in protein expression in placental tissues from women with term and preterm preeclampsia. These proteins are involved in multiple important signaling pathways that regulate growth, angiogenesis, apoptosis, immune response, and stress response. These altered signaling pathways may be responsible for clinical presentations of preeclampsia and fetal growth restriction as the expression levels of some of the proteins are strongly correlated with the clinical symptoms and neonatal body weight. Further study is needed to elucidate the role of these proteins and their associated pathways in the pathogenesis preeclampsia.

## Supporting Information

Table S1 **List of antibodies included in the Protein Pathway Array.**
(DOCX)

File S1 **Normalized data for the Protein Pathway Array.**
(DOCX)
