# Supplementary material for: Protein Profiling of Preeclampsia Placental Tissues
Source: PLoS One. 2014 Nov 13;9(11):e112890. doi: 10.1371/journal.pone.0112890 (PMC4231077; doi:10.1371/journal.pone.0112890)
Supplement: Table S1 — List of antibodies included in the Protein Pathway Array. (DOCX) [file pone.0112890.s001.docx]

| **Supplemental Table 1: List of antibodies included in the Protein Pathway Array** |
| --- |
| **Antibodies specific for phosphorylation** |
| p-PKCα(Ser657), p-PDK1 (Ser241), p-PKCα/βII (Thr638/641),p-P53 (Ser392), p-AKT (Ser473), p-PTEN (Ser380), p-Rb (Ser780), p-β-catenin (Ser33/37/Thr41), p-c-Jun (Ser73), p-STAT 3 (Ser727) **^*^**,p-P44/42 MAPK (Erk1/2) (Thr202/Tyr204)**^*^**, p-GSK-3α/β(Ser21/9), p-P70 S6 Kinase (Thr389), p-eIF4B (Ser422), p-Met (Tyr1234), p-Smad1/5 (Ser463/465), p-ERK5 (Thr218/Tyr220), p-P90RSK (Ser380), p-CREB (Ser133), p-PKCδ(Thr505) **^*^**,p-FAK (Tyr397), p-CDC2 (Tyr15), p-STAT5 (Tyr694), p-Rb (Ser807/811), p-P38MAPK (Thr180/Tyr182). |
| **Antibodies specific for non-phosphorylation** |
| FAS,FOXM1, ERα, Syk, MetRS, twist, Lyn, KLF6,CaMKKα, SK3, Stat1, cyclinB1, cyclinD1, Cdk6, Cdc25B,EGFR**^*^**,Cdk2, p27**^*^**, TDP1, Cdk4, HER2/ErbB2, 14-3-3β**^*^**,PKCα, cyclinE, SLUG, Cdc25C, Hsp90**^*^**, Chk1, MDM2, Cdc2 p34, E2F-1, PCNA, p63, p38α/β, Rap1**^*^**,β-Catenin, p44/42 MAPK (Erk1/2),Akt**^*^**, XIAP, Bcl-2**^*^**, patched, HIF-1α, HIF-2α, TTF-1, p53, Notch4,PTEN, SRC-1, Eg5, HIF-3α, Bax, N-cadherin, TNFα, Cdc42,eIF4B, Vimentin, OPN, Survivin, E-cadherin, TGF-β, ERβ,WT1**^*^**, Mesothelin, VEGF, ATF-1, Ep-CAM, Bad, NF-κB p52, NFκB p50, Calretinin**^*^**,IL-1β, H-Ras, Bcl-6**^*^**, K-Ras, NF-κB p65**^*^**, CREB**^*^**, BID, Maspin, DRG1, Factor XIII B, IGFBP5, HCAM, ICAM-1**^*^**, FLIP_S/L_, PSM**^*^**, Rab 7, VCAM-1, FGF-8,CD10, Bcl-xL, Endoglin**^*^**, Bak, TFIIH p89**^*^**, Nkx-3.1, RIP, nm23-H1/2/3, c-IAP2**^*^**, Epo, PDEF**^*^**,Stat3, ERCC1, uPAR, KAI 1, L-Selectin, PSCA, E-Selectin, AT_1_,ADAM12,IGFBP3,PBEF**^*^**,Nox4**^*^**,Flt-1**^*^**,DLK,PAPP-A,Ebi3**^*^**,HtrA**^*^**,Tie-1,Lipin-2,TFPI-2**^*^**,Alkaline Phosphatase,AnnexinⅪ**^*^**,Heme Oxygenase 1,PIG-S**^*^**,PAF acetylhydrolase 2**^*^**,PSG5,Nrf2**^*^**,PLAC1,TIMP-2,Ptx3**^*^**,PIGF**^*^**,Glypican-3**^*^**,Galectin-13,GPCR2037,p22-phox,PAI-1**^*^**,Choriogonadotropinβ,CD55,COL18A1,JNK1,RhoA,PRMT2. |
